# Associations between the Big Five personality traits, testosterone, and cortisol in adolescent male athletes

**DOI:** 10.5114/biolsport.2024.127390

**Published:** 2023-09-07

**Authors:** Blair T Crewther, Zbigniew Obmiński, Dariusz Turowski, Beata Szczepańska, Helena Mroczkowska

**Affiliations:** 1 Institute of Sport – National Research Institute, Warsaw, Poland; 2 Biomedical Discipline, School of Science and Technology, University of New England, Armidale, Australia

**Keywords:** Androgens, Stress, HPA-axis, Psychology, Neuroendocrine

## Abstract

Testosterone (T) has been conceptualized as a biomarker of individual differences, yet T associations with the Big Five personality traits are inconsistent. Athletes provide a suitable model for evaluation here, as T co-expresses traits related to male-to-male competition and fitness with cortisol (C) playing a moderating role. This study investigated associations between the Big Five traits, T, and C in adolescent male athletes. One hundred and twenty male ice hockey players (aged 14–19 years) were assessed for blood total (T, C) and free (FT, FC) hormones, body-size dimensions (i.e., body mass, height, body mass index [BMI]), the Big Five personality traits (i.e., extraversion, neuroticism, agreeableness, conscientiousness, openness), and trait anxiety. Correlational and regression (with age and BMI as covariates) analyses identified a positive effect of FT on extraversion, but a negative FT effect on neuroticism and anxiety (*p* < 0.05). Significant FT × FC interactions emerged for extraversion and agreeableness. Slope testing revealed that FT had a positive effect on extraversion at the FC mean and +1 SD, and a negative effect on agreeableness with FC at +1 SD. In conclusion, adolescent male athletes with a higher serum FT concentration tended to express higher extraversion, but lower neuroticism and anxiety. The FT association with extraversion was moderated by FC concentration, as was agreeableness. Therefore, high-FT athletes presented a behavioural disposition that favours dominance and resiliency, with some dependencies on FC availability. Since all association effect sizes were weak, replicate studies on larger adolescent samples are needed.

## INTRODUCTION

The androgen testosterone (T) has an established role in sport and exercise, with basal, exercise-induced, and adaptive shifts in T regulating the structural and functional development of the brain and neuromuscular system [1]. Unsurprisingly, T has been conceptualized as biological marker of individual differences from a behavioral perspective, exhibiting high stability over time [2] with a substantial genetic component [3]. As such, personality studies have attempted to identify a functional role for individual T differences, particularly with respect to the Big Five personality traits. While there are some reports of high-T men scoring higher on extraversion [4, 5, 6, 7], others have found a negative association [8, 9] or no relationship between these factors [2, 10]. This inconsistency extends to the remaining Big Five traits of neuroticism, openness to experience, agreeableness, and conscientiousness [2, 5, 8, 9, 10],

From an evolutionary perspective, T helps coordinate the co-expression of traits related to male-to-male competition, fitness, and reproductive success [11]. Therefore, athletic men may provide a more suitable model for evaluating the T relationship with status-relevant personality traits. Indeed, research has shown high-T male athletes exhibit trait behaviours (i.e., more extraversion, less neuroticism and anxiety) [12, 13] that favour dominance and stress resiliency. No such data exists on male athletes during adolescence, a key developmental period characterized by dramatic changes in T production and temporal variation in the Big Five personality dimensions [14, 15]. This is despite suggestions that between 70–85% of successful and unsuccessful athletes can be identified using general measures of personality and mood state [16]; hence, access to this information could enhance sports selection, training preparation, and monitoring among young developing athletes.

The glucocorticoid cortisol (C) also plays a prominent role in sports performance and training adaptation via pleiotropic nervous-system actions that both oppose, and complement, those of T [1, 17]. Another emerging role for C is moderation of the T effect at target tissue. Research on adolescent [18] and adult male athletes [19], for instance, showed that T was a poor independent predictor of physical performance, but significant T linkages to performance were found at different baseline C concentrations. Whether T relationships with personality traits are moderated by C remains unclear. Large meta-analytic studies found no significant T × C effect (a common test for moderation) on the Big Five dimensions [7, 8], or other status-striving traits [7], in adult men. However, the population samples consisted of primarily non-athletic college students, with testing conducted under neutral conditions. To our knowledge, this nuanced T and C interplay with the Big Five traits has not been examined in male athletes of any age.

To address these gaps, a cross-sectional study was conducted to investigate associations between blood hormone (T, C) concentrations and the Big Five personality traits in a homogenous cohort of adolescent male athletes. Consistent with research on athletic men [12, 13], we hypothesized that individual differences in T concentration would be positively associated with extraversion and negatively related to neuroticism. No hypotheses were made with regards to the remaining Big Five traits. We also tested whether C moderates any T and personality relationship, operationally defined as a significant T × C interaction, with follow-up tests to probe the T effect at different C concentrations. Since no comparable data on male athletes exists, we made no firm predictions regarding these associations.

## MATERIALS AND METHODS

### Participants

One hundred and twenty male ice hockey players (aged 14–19 years) were recruited for this study. Athlete pre-screening did not reveal any medical and health problems, including endocrine and psychiatric disorders, that would affect the ability of participants to complete the experimental protocols, as detailed below. The study participants gave written informed consent, after receiving a full explanation of the study goals, procedures, and benefits. For those athletes under 18 years of age, additional consent from a parent or guardian was obtained. Ethical approval (number KEBN-20-54-HM) was granted from the ethics committee at the Institute of Sport – National Research Institute, Warsaw, Poland.

### Study procedures

Data were collected using a cross-sectional design, as part of a national testing programme for the Polish Ice Hockey Federation. During a one-day visit to the laboratory, the participants completed the following assessments (in order) after an overnight fast; blood collection at ~8 am and anthropometric measurements, before breakfast was eaten from ~9–10 am. Testing resumed with an exercise assessment (not part of this study) at around 11–12 am, followed by a full recovery period before lunch was eaten from ~2–3 pm. Finally, questionnaires were self-completed in the afternoon (3–4 pm) to identify the Big Five personality traits and trait anxiety. The selection and order of testing is based on established protocols at the Institute of Sport – National Research Institute. To eliminate the confounding effects of fatigue, the athletes refrained from any physical training in the previous 24 hrs. Test implementation was performed by the same technicians and standardized across participants.

### Blood hormone assessment

A 10 mL sample of venous blood was drawn from the antecubital vein and transferred into a polystyrene tube. The sample was left to clot after collection, before centrifugation (2000 g for 10 mins) to separate the serum component, which was aliquoted into another tube and stored in a -80° C freezer. After thawing and centrifugation, the serum samples were tested in duplicate using commercial enzyme-linked immunoassay (ELISA) kits (DRG, Germany). The lower detection limits for the T and C ELISA kits were 0.69 nmol · L^-1^ and 5.5 nmol · L^-1^, respectively. Sample testing was extended to include the blood measurement of free C (FC) and free T (FT) concentrations, as they represent the smaller portion of each steroid (1–10% of total hormones) that is biologically active at target tissue [20].

To determine serum FC concentration, expressed in nmol · L^-1^, we used a Centrifree YM-30 ultrafiltration device (Millipore, USA) with a 30kDa cut-off. Briefly, the loaded sample was centrifuged (2000 g for 30 mins at 37°C) to isolate the steroid free portion in sample filtrate. Following the centrifugation procedure, the filtrate was assayed using an ELISA kit for salivary C (DRG, Germany) with a detection limit of 0.25 nmol · L^-1^. Serum FT concentration, expressed in pmol · L^-1^, was estimated from the concentration measures of T and sex-hormone binding globulin (SHBG) using a validated algorithm [21], whereby FT concentration = 24.00314 × T/Log10(SHBG) – 0.04599 × T^2^. Serum SHBG concentration was determined using a commercial ELISA kit (DRG, Germany) that had a detection limit of 4 nmol · L^-1^. The inter-assay kit CV’s, based on internal controls, were less than 5% on average across all blood T, C, and SHBG assays, and the salivary C assay.

### Anthropometric testing

Participant body mass was measured to the nearest 0.1 kg using digital scales (Tanita, Japan). Standing height was assessed to the nearest 1 cm with a freestanding stadiometer (Siber-Hegner, Switzerland). The participants wore shorts and a shirt, without shoes and socks, during this evaluation. As a general indicator of health status, we computed a body mass index (BMI) for each participant by dividing body mass by height (expressed in m^2^).

### Assessment of personality traits

The NEO-FFI Personality Questionnaire was chosen to examine the athletes’ personality type with regards to the Big Five factor model [22]. The NEO-FFI Personality Questionnaire consists of 60 items that measure the five-factor model (12 items each) of: (1) neuroticism, (2) extraversion, (3) openness to experience, (4) agreeableness, and (5) conscientiousness. Trait anxiety was also measured, being a standard assessment in our laboratory, and one that correlates positively with neuroticism and/or negatively with extraversion among male athletes [12, 13]. The 20-item version of the Spielberger State-Trait Anxiety Inventory (STAI) was selected for this purpose [23]. Both inventories were completed in pen and paper format using the translated (English into Polish) versions of the NEO-FFI [24] and STAI [25].

### Statistical procedures

The study data were prepared and analyzed in the R (version 4.1.1) programming environment [26] using several packages (i.e., easystats, readxl, psych, ggplot2, interactions, sjPlot). In the first instance, descriptive means and dispersion statistics were calculated for all demographic, body size, hormonal, and personality variables. Next, the zero-order, linear associations between variables were explored using Pearson correlations. Based on standard conventions, these correlations (absolute values) can be interpreted as being either a weak (0.10 to < 0.30), moderate (0.30 to < 0.50) or strong effect (0.50 to 1.00). The correlational results were also used to identify, and subsequently remove, strongly-related variables to avoid multicollinearity in the main regression analyses.

To determine if individual differences in T and C concentrations were related to each personality trait, we ran a series of hierarchical regression models. Each model was implemented as a two-step process. In step one, FT and FC were entered as predictors with age and BMI as covariates. In step two, the FT × FC interaction was entered into the model. We report all significant effects with an effect-size correlation (i.e., partial *r*), computed from model t values and degrees of freedom [27]. Following a significant interaction, simple slopes were examined for three values of the moderator: -1 SD, sample mean, and +1 SD [28]. Further probing was performed using Johnson-Neyman interval plots to identify the regions of significance [29]. Statistical significance was set at an alpha level of *p* ≤ 0.05. Given that T x C personality relationships are generally underpowered [7], we applied a lower alpha threshold (*p* ≤ 0.10) to establish a significant interaction. All assumptions of regression modeling were met, based on visual checks of model residuals.

## RESULTS

Descriptive statistics for all variables are presented in Table 1. Correlational testing revealed positive relationships (weak to strong effects) between most body-size indicators, with BMI also found to be negatively related (weak effect) to FC concentration (all *p* < 0.05). The serum T and FT concentration measures were strongly and positively related, as were serum C and FC (*p* < 0.001). Both serum T and FT concentrations were negatively related to neuroticism and anxiety, but positively related to extraversion (*p* < 0.05), with weak effect sizes on these bivariate comparisons. Significant interrelationships also emerged, both positive and negative, between the Big Five personality dimensions and/or trait anxiety. These associations were generally weak, apart from a strong positive relationship between neuroticism and anxiety (*p* < 0.001).

**TABLE 1:** Descriptive results for each variable and zero-order correlations between variables.

Variables	Mean	Range	1	2	3	4	5	6	7	8	9	10	11	12	13	14
1. Age (years)	16.7	14.0 – 18.6	1	-0.06	**0.20**	**0.28**	0.12	0.08	0.00	-0.01	0.06	-0.03	0.04	**0.19**	0.03	-0.05
2. Height (m)	1.79	1.64 – 1.93		1	**0.51**	-0.07	-0.12	-0.10	0.02	0.00	0.02	-0.01	0.07	-0.02	0.01	0.12
3. Body mass (kg)	73.8	53.1 – 93.4			1	**0.82**	-0.04	-0.01	-0.15	-0.16	-0.04	-0.01	0.04	0.06	0.06	0.03
4. BMI (kg · m^2^)	23.1	17.6 – 28.2				1	0.04	0.06	-0.17	**-0.18**	-0.07	0.01	0.01	0.08	0.07	-0.05
5. Testosterone (nmol · L^-1^)	22.5	9.4 – 38.5					1	**0.92**	0.16	0.11	**-0.20**	**0.21**	0.09	-0.09	0.15	**-0.21**
6. FT (pmol · L^-1^)	342	131 – 686						1	0.16	0.08	**-0.23**	**0.23**	0.11	-0.06	0.16	**-0.25**
7. Cortisol (nmol · L^-1^)	518	264 – 835							1	**0.92**	-0.15	0.09	0.13	0.04	0.13	-0.18
8. FC (nmol · L^-1^)	63.8	17.9 – 215								1	-0.12	0.07	0.11	0.04	0.12	-0.13
9. Neuroticism (score)	20.7	2 – 42									1	**-0.41**	-0.01	-0.08	**-0.33**	**0.75**
10. Extraversion (score)	31.3	12 – 44										1	**0.23**	0.03	**0.48**	**-0.39**
11. Openness (score)	24.4	11 – 37											1	-0.12	0.16	-0.05
12. Agreeableness (score)	28.4	16 – 44												1	**0.42**	-0.07
13. Conscientiousness (score)	35.4	13 – 47													1	**-0.28**
14. Anxiety (score)	39.5	25 – 60														1

Key: BMI = body mass index, FT = free testosterone, FC = free cortisol. Significant correlations are highlighted in bold.

The stage one regression models (see Table 2) yielded a significant negative effect of FT concentration on both neuroticism (partial *r* = -0.23) and anxiety (partial *r* = -0.23), along with a significant positive effect of FT on extraversion (partial *r* = 0.23), when controlling for other predictors and covariates. We found no significant FT relationships with openness, agreeableness, and conscientiousness at this level of analyses, whilst FC concentration was unrelated (all *p* > 0.179) to all personality traits. The fitted models were weak, explaining only 1–5% of personality trait variation, and only the neuroticism and anxiety predictions were significant. Stage two revealed significant FT × FC interactions when predicting extraversion (*p* = 0.079, partial *r* = 0.16) and agreeableness (*p* = 0.030, partial *r* = -0.20), once all lower-order main effects were controlled for. The fitted models were again relatively weak (1–5% variation explained) and largely non-significant, except for neuroticism and anxiety, and only agreeableness showed a significantly better fit (ΔR^2^ = 3.2%) with the FT × FC term included.

**TABLE 2:** Hierarchical regression models predicting the Big Five personality traits and trait anxiety.

Predictors	Neuroticism		Extraversion		Openness		Agreeableness		Conscientiousness		Anxiety	

	Est.	Est.	Est.	Est.	Est.	Est.	Est.	Est.	Est.	Est.	Est.	Est.
Age	1.111 (0.993)	1.098 (0.992)	-0.418 (0.756)	-0.403 (0.749)	0.205 (0.628)	0.214 (0.626)	1.219* (0.611)	1.201* (0.601)	-0.086 (0.789)	-0.086 (0.792)	-0.185 (0.952)	-0.195 (0.953)
BMI	0.379 (0.340)	-0.374 (0.340)	0.049 (0.259)	0.043 (0.257)	0.038 (0.215)	0.035 (0.215)	0.089 (0.209)	0.094 (0.206)	0.244 (0.270)	0.244 (0.272)	-0.189 (0.326)	-0.186 (0.327)
FT	-0.020* (0.008)	-0.001 (0.018)	0.015* (0.006)	-0.007 (0.014)	0.005 (0.005)	-0.008 (0.011)	-0.005 (0.005)	0.017 (0.011)	0.010 (0.006)	0.011 (0.014)	-0.020** (0.008)	-0.006 (0.017)
FC	-0.027 (0.021)	0.066 (0.084)	0.008 (0.016)	-0.100 (0.063)	0.014 (0.013)	-0.049 (0.053)	0.007 (0.013)	0.115* (0.051)	0.022 (0.016)	0.026 (0.067)	-0.027 (0.020)	0.041 (0.080)
FT × FC		-0.000 (0.000)		0.000# (0.000)		0.000 (0.000)		-0.000* (0.000)		-0.000 (0.000)		-0.000 (0.000)

R^2^ adjusted	0.049*	0.051*	0.024	0.041	0.011	0.007	0.014	0.046	0.012	0.004	0.045*	0.043
ΔR^2^ adjusted		0.002		0.017		-0.004		0.032*		-0.008		-0.002

Note: Estimates are shown with standard errors (). BMI = body mass index, FT = free testosterone, FC = free cortisol. Significance levels are depicted as ^#^ *p* ≤ *0.10*

* *p* ≤ *0.05*

** *p* ≤ *0.01.*

**FIG. 1:**
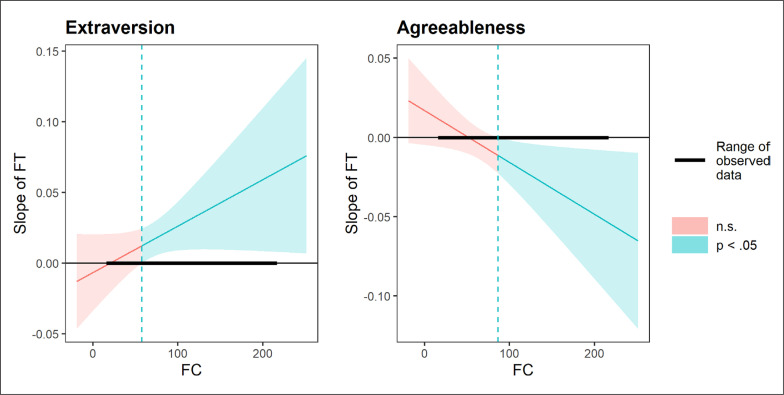
Interactions between free testosterone (FT) and free cortisol (FC) concentrations in relation to extraversion and agreeableness.

When probing the FT × FC effect on extraversion, slope testing revealed a FT relationship at the FC mean (estimate = 0.014, SE = 0.006, *p* = 0.019) and +1 SD (estimate = 0.026, SE = 0.009, *p* = 0.003), but not at -1 SD (estimate = 0.002, SE = 0.009, *p* = 0.817). The Johnson-Neyman plot confirmed a positive and significant FT link to extraversion, but only when the FC interval was between 57.8 and 441.8 nmol · L^-1^ (Figure 1). Regarding agreeableness, simple slopes identified a FT relationship at a high (+1 SD) FC concentration (estimate = -0.016, SE = 0.007, *p* = 0.026), but not at the sample mean (estimate = -0.004, SE = 0.005, *p* = 0.427) or -1 SD (estimate = 0.008, SE = 0.008, *p* = 0.281). The Johnson-Neyman plot confirmed a significant negative effect of FT on agreeableness, but only when FC concentration was outside the interval of -104.3 to 86.5 nmol · L^-1^ (Figure 1).

As a robustness check, all regression models were repeated, but with the age and BMI covariates removed: see supplement Table S1. The emergence of significant FT associations with neuroticism (partial *r* = -0.23), extraversion (partial *r* = 0.22), and anxiety (partial *r* = -0.24) paralleled our main findings, as did a significant FT × FC effect on extraversion (partial *r* = 0.17) and agreeableness (partial *r* = -0.20). Follow-up analyses with simple slopes (Table S2) and Johnson-Neyman plots (Figure S1) revealed trends and regions of significance that are coherent with our initial results. Although the fitted outcomes were still weak in strength (1–6% explained variance), the explanatory models for neuroticism, extraversion and anxiety were all significant after covariate removal. Once again, only agreeableness showed a significantly better fit (ΔR^2^ = 3.1%) with inclusion of the FT × FC interaction.

## DISCUSSION

The purpose of this study was to investigate associations between the Big Five personality traits and morning blood T and C concentrations in adolescent male athletes. Complementary sets of analyses verified that individual differences in serum FT concentration were positively associated with extraversion, but negatively related to neuroticism and anxiety. No link between FC and any personality trait was, however, seen. Complex interplay also transpired for extraversion and agreeableness, with FT being positively related to extraversion and negatively related to agreeableness at a mean or higher FC concentration.

As hypothesized, adolescent males with an elevated serum FT concentration tended to express a higher level of extraversion, along with less neuroticism and anxiety. These findings parallel research on male athletes [12, 13], adult men [4, 6, 7], and mixed-sex cohorts [5]. For high-T males, higher scores on extraversion, coupled with lower neuroticism and anxiety, could reflect a general disposition for emotional stability, less tension, as well as stronger self-efficacy, positive affect, and motivation; all facets of dominance and stress resiliency. Nonetheless, it’s not clear why T might preferentially relate to these dimensions and not others. One possibility is adaptive calibration of selected traits necessary for athletic performance. For example, stronger FT associations with training motivation and competitiveness were seen in elite than non-elite performers [30, 31, 32]. Our results could also reflect study specificities, such as the targeting of young talented athletes in a dominance-related sport, and data collection across a full day of physiological and psychological testing. The possible role of age-specific effects of T on the Big Five traits [14] is another open question. A replicate study on adolescent males representing different levels of athletic competition (i.e., elites, non-elites, controls), and varying age categories, would help answer these questions.

Serum FC concentration did not correlate with, or predict, any Big Five personality dimension or trait anxiety in our sample of adolescent athletes. Athlete studies in this area are equivocal, with reports of positive salivary FC linkages to some (i.e., extraversion, conscientiousness), but not all, of the Big Five personality traits [33, 34], while others found no serum C associations with neuroticism, extraversion or anxiety [12, 13]. This incongruency is perhaps not surprising, given reports of null or inconsistent results for the C or FC biomarkers are widespread in the general population [7, 8, 10, 35, 36, 37, 38]. These inconclusive findings extend to other hormones of the hypothalamic-pituitary-adrenal (HPA) axis (e.g., adrenocorticotropic hormone) and their relationship with the Big Five personality traits [39]. This equivocality could be attributed to study heterogeneity, in terms of differences in sample composition (e.g., blood, saliva, hair) and timing, the indices of HPA activity used (e.g., morning versus afternoon C, cumulative C, C awakening response), and assay procedures. An alternative viewpoint is that C, as a stress biomarker exerting pleiotropic actions on different target tissue and time scales [1, 17], functionally interacts with other biomarkers in such a way that independent testing of this variable fails to capture.

Finally, and as noted earlier, nuanced hormonal interactions emerged in relation to extraversion and agreeableness, with a higher FT concentration associated with more extraversion and less agreeableness when FC exceeded 57.8 nmol · L^-1^ and 86.5 nmol · L^-1^ respectively, which equates to the 55^th^ and 78^th^ percentile scores for FC. The latter result is reasonably consistent with work on androgen-deprived adult men, who reported higher agreeableness than controls [40]. Comparable data on adolescent athletic males does not exist; nevertheless, it may be considered that low agreeableness (among high-FT adolescent males) might not be viewed negatively in the context of athlete testing where cooperation and empathy are less relevant. Other T-connected traits, like physical fitness, have shown similar dependencies on C. For instance, salivary FC concentration moderated the FT effect on exercise performance in both male weightlifters [19] and ice hockey players [18]. Taken together, these results suggests that elevated FC secretion might be an important preconditioning factor, at least for athletic men and adolescent males. This position would also explain the lack of significant T × C interactions with the Big Five personality traits in larger studies on, primarily, college age students [7, 8]. Consequently, we propose that the presence of T (or FT) x C (or FC) interactions is contingent on many factors, like the studied population and personality trait measured, environmental or situational conditions, and potentially other (e.g., vitamin 25 [OH]D) steroid biomarkers [18].

Given the high proportion of successful and unsuccessful athletes that can be identified using general personality and mood state measures [16], we propose developing a psychological inventory for young male athletes that includes those Big Five personality traits deemed relevant to different team sports [41]. This information could form part of a broader testing strategy for talent identification, training and competition preparation, and seasonal monitoring. As one example, knowledge of behavioral tendencies might be used to assign mental training techniques, team communication, and tactical thinking skills to produce positive individual and team outcomes [41]. Supplementing this information with blood FT and FC data, or salivary FT and FC as a non-invasive alternative, could provide further insight regarding how a personality and biological disposition might affect athlete behavior in sport. With the advent of rapid hormone diagnostics [42, 43], it may be possible to intervene in relative real-time to promote a hormonal profile that exploits these psychological differences for athletic gain; see work on preconditioning strategies in sport [44]. Since sporting context can differentially activate T and C secretion, whilst inducing transient shifts in behavior, the concurrent assessment of relevant states (e.g., competitive state anxiety, situational motivation) would be prudent to capture context-related changes in these hormone-personality-behavior associations and thus, help refine the strategies prescribed.

One strength of this study is cohort homogeneity, although this does limit our ability to make broader conclusions for other athletic groups and non-athletes. Moreover, the effect sizes for the hormone-personality associations were weak and the current sample is considered small for testing interactions [7]. Further bias might arise from the time difference between the hormonal (morning) and personality (afternoon) assessment. Data collected within our laboratory does indicate that FT and FC measured in saliva are highly stable (Spearman *r* = 0.87–0.97) across the day (i.e., rank order is maintained), so we anticipated little bias in the modeled results. Temporal variation in the Big Five personality dimensions, from childhood up to early adulthood [14, 15], adds another level of complexity when attempting to characterize these linkages; however, it is known that trait stability remains relatively high across adolescence [15]. Finally, we acknowledge that T and C secretion can vary on a moment-to-moment basis, due to a myriad of situational and environmental cues in sport. Hence, a longitudinal study on a larger adolescent cohort is needed to ascertain the robustness of our findings across different contextual settings.

## CONCLUSIONS

Adolescent male athletes with a higher serum FT concentration showed a tendency for higher extraversion, lower neuroticism, and less anxiety. The FT relationship with extraversion also depended on serum FC concentration, as did agreeableness. Therefore, high-FT athletes in this study displayed personality tendencies likely to favour dominance and stress resiliency, although complicated by more nuanced FT and FC interplay. These findings must be interpreted in light of weak association effect sizes and a lack of comparable research, which underscores the need for study replication on larger athlete samples.

## Supplementary Material

Associations between the Big Five personality traits, testosterone, and cortisol in adolescent male athletes

## Data Availability

The data is not publicly available, due to ethical restrictions.
