# Supplementary material for: Associations between the Big Five personality traits, testosterone, and cortisol in adolescent male athletes
Source: Biol Sport. 2023 Sep 7;41(1):279–86. doi: 10.5114/biolsport.2024.127390 (PMC10765448; doi:10.5114/biolsport.2024.127390)
Supplement: Associations between the Big Five personality traits, testosterone, and cortisol in adolescent male athletes [file JBS-41-50674-s1.pdf]

Supplemental Materials.

TABLE S1. Hierarchical regression models predicting the Big Five personality traits and trait anxiety.

Predictors	Neuroticism Est.	Neuroticism Est.	Extraversion Est.	Extraversion Est.	Openness Est.	Openness Est.	Agreeableness Est.	Agreeableness Est.	Conscientiousness Est.	Conscientiousness Est.	Anxiety Est.	Anxiety Est.
FT	-0.020* (0.008)	-0.001 (0.018)	0.015* (0.006)	-0.007 (0.014)	0.005 (0.005)	-0.007 (0.011)	-0.004 (0.005)	0.018 (0.011)	0.011 (0.006)	0.011 (0.014)	-0.020** (0.008)	-0.006 (0.017)
FC	-0.023 (0.020)	0.072 (0.083)	0.008 (0.015)	-0.101 (0.062)	0.014 (0.013)	-0.050 (0.052)	0.006 (0.013)	0.114* (0.051)	0.019 (0.016)	0.023 (0.066)	-0.024 (0.019)	0.044 (0.080)
FT ´ FC		-0.000 (0.000)		0.000# (0.000)		0.000 (0.000)		-0.000* (0.000)		-0.000 (0.000)		-0.000 (0.000)
R^2 adjusted	0.049*	0.052*	0.038*	0.056*	0.004	0.009	-0.011	0.020	0.022	0.014	0.058*	0.056*
ΔR^2 adjusted		0.003		0.018		0.005		0.031*		-0.008		-0.002

Note: Estimates are shown with standard errors (). FT = free testosterone, FC = free cortisol. Significance levels are depicted as # *p ≤ 0.10 * p ≤ 0.05 ** p ≤ 0.01*

TABLE S2. Simple slope analyses for free testosterone (FT) and free cortisol (FC) concentrations in relation to extraversion and agreeableness.

Extraversion				Agreeableness			
Slope of FT when FC = 27.10 (-1 SD):				Slope of FT when FC = 27.10 (-1 SD):			
Est.	SE	t value	*p* value	Est.	SE	t value	*p* value
0.002	0.009	0.208	0.835	0.009	0.008	1.212	0.228
Slope of FT when FC = 63.83 (Mean):				Slope of FT when FC = 63.83 (Mean):			
Est.	SE	t value	*p* value	Est.	SE	t value	*p* value
0.014	0.006	2.367	0.020	-0.003	0.005	-0.583	0.561
Slope of FT when FC = 100.56 (+ 1 SD):				Slope of FT when FC = 100.56 (+ 1 SD):			
Est.	SE	t value	*p* value	Est.	SE	t value	*p* value
0.026	0.009	3.018	0.003	-0.015	0.007	-2.100	0.038

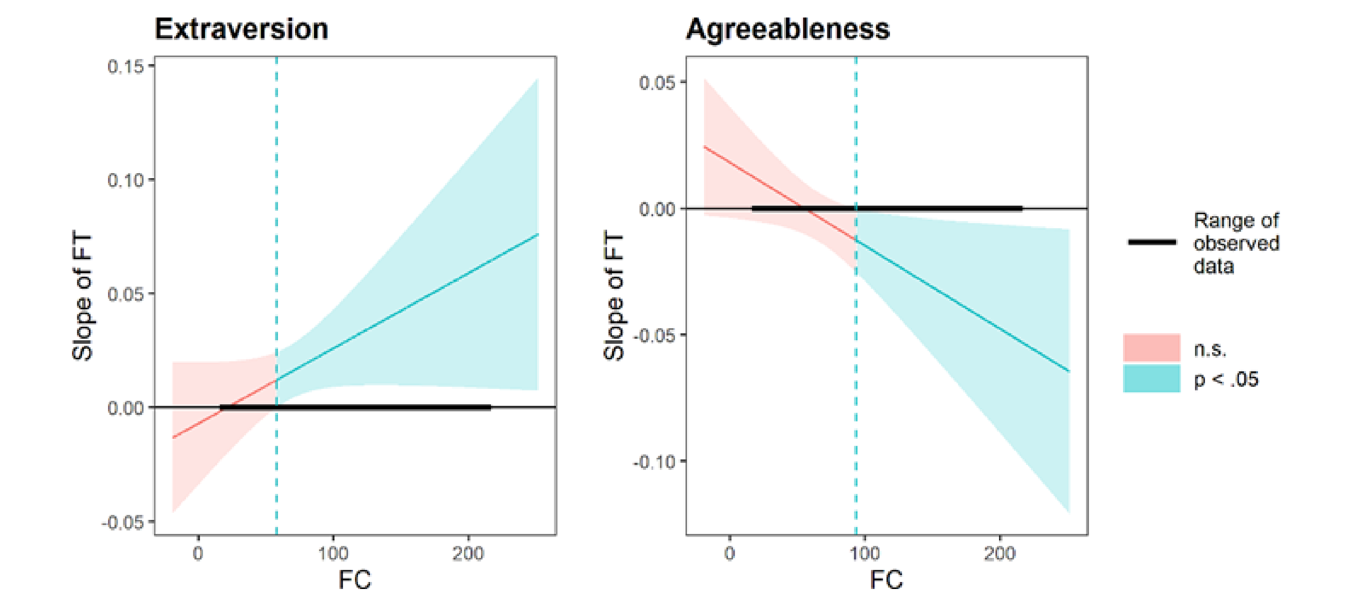

FIG. S1. Interactions between free testosterone (FT) and free cortisol (FC) concentrations in relation to extraversion and agreeableness.
